# Migration background and diabetes technology use at diagnosis and follow-up in children and adolescents with type 1 diabetes: a single-centre study

**DOI:** 10.1007/s00592-026-02758-6

**Published:** 2026-07-29

**Authors:** Dagmar Meraner, Marjan Arvandi, Katharina Pajk, Eva Vonmetz, Wilhelm Oberaigner, Veronika Haslwanter, Sibylle Puntscher, Elisabeth Binder, Elisabeth Steichen, Uwe Siebert, Ines Schaffer, Sabine E. Hofer, Daniela Schmid

**Affiliations:** 1 Department of Pediatrics 1, Medical University of Innsbruck, Innsbruck, 6020 Austria; 2 Institute of Public Health, Medical Decision Making and Health Technology Assessment, Department of Public Health, Health Services Research and Health Technology Assessment, UMIT TIROL - University for Health Sciences and Technology, Eduard-Wallnoefer-Zentrum 1, Hall in Tirol, 6060 Austria; 3 Center for Health Decision Science, Departments of Epidemiology and Health Policy & Management, Harvard T.H. Chan School of Public Health, Boston, MA USA; 4 Program on Cardiovascular Research, Institute for Technology Assessment and Department of Radiology, Harvard Medical School, Massachusetts General Hospital, Boston, MA USA; 5 Faculty of Life Sciences, Albstadt-Sigmaringen University, Sigmaringen, Germany

**Keywords:** Type 1 diabetes, Migration background, Insulin pump, Automated insulin delivery, Pediatric diabetes

## Abstract

**Aims:**

To investigate whether migration background is associated with diabetes technology use at diagnosis and during follow-up in children and adolescents with type 1 diabetes.

**Methods:**

This cross-sectional study included children and adolescents aged 4–19 years with type 1 diabetes duration ≥ 1 year. Insulin pump therapy at diagnosis and diabetes technology use at follow-up (defined as insulin pump therapy or automated insulin delivery [AID]) were compared between participants with and without migration background. Multivariable logistic regression was used to assess factors associated with technology use.

**Results:**

A total of 143 participants (mean age 13.2 ± 3.7 years) were included, of whom 24.5% had a migration background. At diabetes onset, insulin pump therapy was less frequent in participants with migration background compared to those without (28.6% vs. 50.0%, *p* = 0.027). At follow-up, diabetes technology use was numerically lower in participants with migration background (60.0% vs. 76.8%); however, the difference did not reach statistical significance (*p* = 0.052). In adjusted analyses, migration background was not significantly associated with diabetes technology use at follow-up (OR 2.33, 95% CI 0.92–5.89), whereas younger age and longer diabetes duration were independently associated with technology use.

**Conclusions:**

Children with migration background were less likely to receive insulin pump therapy at diagnosis. At follow-up, differences in diabetes technology use were smaller and not statistically significant. These findings are consistent with the hypothesis of delayed technology uptake, although longitudinal studies are required to determine the timing of diabetes technology initiation.

**Supplementary Information:**

The online version contains supplementary material available at https://doi.org/10.1007/s00592-026-02758-6.

## Introduction

 The incidence of type 1 diabetes (T1D) has increased worldwide and across Europe over recent decades, with similar trends observed in Austria [1–3]. At the same time, migration has become an increasingly important demographic factor in many European countries, including Austria [4].

Several studies have shown that children and adolescents with T1D and a migration background (MB) have poorer metabolic outcomes and higher rates of acute complications, including diabetic ketoacidosis and severe hypoglycaemia [5–9]. In addition, lower uptake of diabetes technology, such as insulin pumps and continuous glucose monitoring, has been reported in individuals with MB [10–12]. Given the well-established benefits of insulin pump therapy and automated insulin delivery (AID) systems for glycaemic control and quality of life, equitable access to diabetes technology has become an important indicator of high-quality diabetes care.

Barriers to healthcare access in populations with MB may include language difficulties, cultural differences and unfamiliarity with the healthcare system [13]. These factors may particularly affect access to advanced diabetes technologies, which require structured education and early integration into diabetes care.

Although disparities in diabetes technology use have been described previously, it remains unclear whether these differences are already present at the time of diagnosis or become apparent later during routine diabetes care. Distinguishing between these scenarios is clinically relevant because it may help identify the most appropriate time points for targeted interventions to improve equitable access to diabetes technology.

To address this knowledge gap, this study aimed to investigate whether migration background was associated with insulin pump therapy at diabetes diagnosis and diabetes technology use at follow-up in children and adolescents with T1D treated at a paediatric diabetes centre in Austria.

## Methods

### Patients and data collection

This single-centre, cross-sectional observational study consecutively enrolled children and adolescents with type 1 diabetes (T1D) who were regularly followed at the paediatric diabetes centre at the Medical University of Innsbruck, Austria. The centre provides care for the majority of paediatric T1D cases in Tyrol/Western Austria.

Participants were recruited over a six-month period from January to June 2023. Inclusion criteria were age between 4 and 19 years and a T1D duration of at least one year. Patients with other types of diabetes were excluded.

A total of 163 patients met the inclusion criteria, of whom 8 declined participation. Of the 155 enrolled participants, 12 were excluded due to missing information on migration background, resulting in a final sample of 143 participants.

Migration background (MB) was defined according to Statistics Austria as both parents being born outside Austria. Participants without MB had at least one parent born in Austria. Individuals with MB were further classified as first-generation (patient and parents born abroad) or second-generation (parents born abroad, patient born in Austria) [4, 14].

Data at diabetes onset included age, sex, HbA1c, pH, insulin therapy (insulin pump or multiple daily injections), and glucose monitoring method (self-monitoring of blood glucose or continuous glucose monitoring [CGM]). Because automated insulin delivery (AID) systems were not available at diabetes onset during the study period, insulin pump therapy alone was considered the diabetes technology measure at diagnosis. At follow-up, diabetes technology use was defined as insulin pump therapy or AID.

At the study visit, data collected included current age, diabetes duration, HbA1c, insulin therapy (insulin pump, automated insulin delivery [AID], or multiple daily injections), glucose monitoring method, and acute complications since diabetes diagnosis (diabetic ketoacidosis and severe hypoglycaemia).

Information on migration history and household language was obtained using a structured questionnaire and a semi-structured interview administered at the study visit. To minimise selection bias, all eligible patients attending routine follow-up visits during the recruitment period were consecutively invited to participate.

### Statistical analysis

Continuous variables are presented as mean ± standard deviation (SD) and were compared using unpaired t-tests. Categorical variables are presented as frequencies and percentages and were compared using Chi-square or Fisher’s exact tests, as appropriate.

The Cochran–Armitage trend test was used to assess trends in diabetes treatment according to migration background.

Multivariable logistic regression analyses were performed to assess factors associated with diabetes technology (insulin pump or AID systems) use at follow-up. Models were adjusted for age, sex, and diabetes duration. The regression analyses were based on complete data for all included model variables. All 143 participants were included in the logistic regression analysis. Complete data were available for all variables included in the model (migration background, age, diabetes duration, and sex).

Given the limited sample size and potential for sparse data, Firth logistic regression was applied as a sensitivity analysis. Household language was analysed in a separate model. No adjustment for multiple comparisons was performed. Therefore, p-values should be interpreted as exploratory, and the findings should be considered hypothesis-generating.

All analyses were performed using SAS version 9.4, and statistical significance was defined as *p* < 0.05. Data were anonymised prior to analysis.

## Results

### Study population

A total of 143 children and adolescents with type 1 diabetes were included, of whom 24.5% (*n* = 35) had a migration background (MB). Baseline characteristics are shown in Table 1.

**Table 1:** Baseline characteristics of the study population stratified by migration background

	non-MB *N* = 108	MB *N* = 35	*P*-value
Female, *n* (%)	46 (42.6)	18 (51.4)	0.361
Age at diabetes onset, years (mean ± SD)	6.3 ± 3.8	7.4 ± 3.0	0.126
Age at study visit, years (mean ± SD)	13.1 ± 3.5	13.2 ± 3.8	0.850
Diabetes duration, years (mean ± SD)	6.8 ± 4.0	5.6 ± 2.5	0.040
HbA1c at diagnosis, % (mmol/mol), (mean ± SD)	11.4% ± 2.2% (101)	11.9% ± 1.9% (107)	0.256

Data are presented as number (percentage) or mean ± standard deviation (SD). Differences between groups were assessed using chi-square or Fisher’s exact tests for categorical variables and unpaired t-tests for continuous variables

*non-MB* without migration background, *MB* with migration background, *HbA1c* glycated haemoglobin

No significant differences were observed between participants with and without MB in terms of age at diabetes onset, age at study visit, or sex distribution. Diabetes duration was shorter in participants with MB (5.6 ± 2.5 years) compared to those without MB (6.8 ± 4.0 years; *p* = 0.040).

### Diabetes technology use at diagnosis and follow-up

#### Insulin pump therapy at diagnosis

**Fig. 1:**
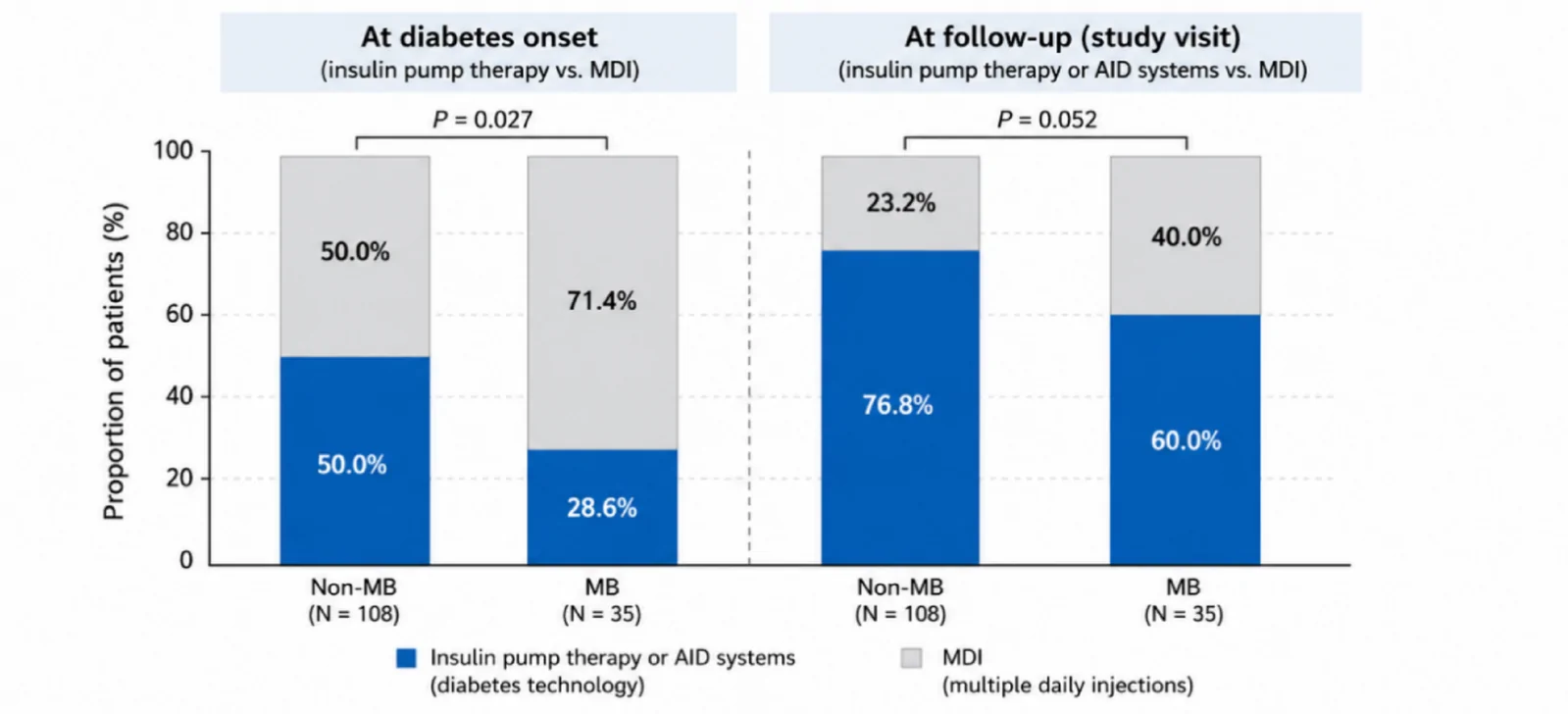
Diabetes technology use at diabetes onset and at follow-up stratified by migration background. At diabetes onset, insulin pump therapy was compared with multiple daily injections (MDI). At follow-up (study visit), diabetes technology use is presented as insulin pump therapy or automated insulin delivery (AID) systems versus MDI. Differences between groups were more pronounced at diabetes onset and less pronounced at follow-up. Data are presented as percentages. *P*-values were calculated using chi-square tests. *non-MB* without migration background, *MB* with migration background, *AID* automated insulin delivery, *MDI* multiple daily injections

At diabetes onset, insulin pump therapy was used in 50.0% of participants without MB and 28.6% of those with MB (*p* = 0.027) (Table 2; Fig. 1).

**Table 2:** Diabetes treatment, metabolic outcomes, and acute complications at diabetes onset and follow-up stratified by migration background

	non-MB *N* = 108	MB *N* = 35	*P*-value
Diabetes onset			
Insulin pump therapy, *n* (%), (*n*_missing_ = 2)	53 (50.0)	10 (28.6)	0.027
MDI, *n* (%), (*n*_missing_ = 2)	53 (50.0)	25 (71.4)	
CGM use, *n* (%), (*n*_missing_ = 7)	27 (26.2)	8 (24.2)	0.822
Follow-up (study visit)			
Diabetes technology (insulin pump or AID), *n* (%)	83 (76.8)	21 (60.0)	0.052
Multiple daily injections (MDI), *n* (%)	25 (23.2)	14 (40.0)	
Insulin pump therapy, *n* (%)	41 (38.0)	11 (31.4)	0.147
Automated insulin delivery (AID), *n* (%)	42 (38.8)	10 (28.6)	
CGM use, *n* (%)	102 (94.4)	30 (85.7)	
HbA1c, % (mmol/mol)	7.6% ± 1.5% (60)	8.0% ± 1.8% (64)	0.181
HbA1c ≤ 7.0% (53 mmol/mol), *n* (%)	45 (41.7)	11 (31.4)	0.281
Acute complications			
DKA at onset (pH < 7.30), *n* (%), *n*_missing_ = 12	51 (51.0)	14 (45.0)	0.629
DKA since diagnosis, *n* (%)	21 (19.7)	10 (28.6)	0.497
Severe hypoglycaemia since diagnosis, *n* (%)	9 (8.4)	5 (14.3)	0.312

Data are presented as number (percentage) or mean ± standard deviation (SD). Differences between groups were assessed using chi-square or Fisher’s exact tests for categorical variables and unpaired *t*-tests for continuous variables, as appropriate

*non-MB* without migration background, *MB* with migration background, *AID* automated insulin delivery, *MDI* multiple daily injections, *CGM* continuous glucose monitoring, *DKA* diabetic ketoacidosis, *HbA1c* glycated haemoglobin

#### Diabetes technology use at follow-up

At the study visit, 76.8% of participants without MB and 60.0% of those with MB were treated with diabetes technology (insulin pump or automated insulin delivery [AID] systems), while the remainder received multiple daily injections (MDI) (*p* = 0.052) (Table 2; Fig. 1). When treatment modalities were analysed separately (insulin pump, AID, MDI), no statistically significant differences were observed between groups (*p* = 0.147).

The numerical difference in diabetes technology use between participants with and without MB was smaller at follow-up than at diagnosis.

### Multivariable analysis

**Fig. 2:**
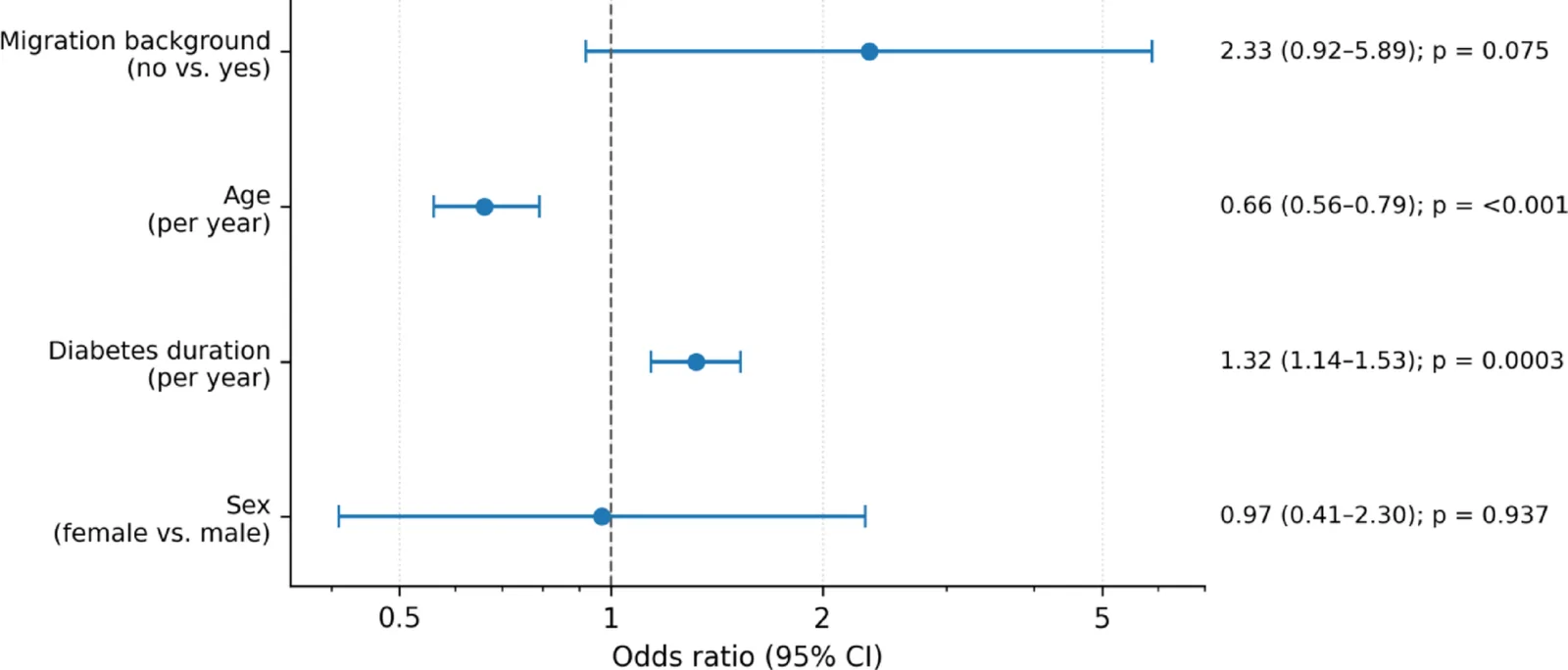
Factors associated with diabetes technology use at follow-up (multivariable logistic regression). Odds ratios (ORs) with 95% confidence intervals (CIs) are shown. The model was adjusted for migration background, age, sex, and diabetes duration. *OR* odds ratio, *CI* confidence interval

In unadjusted analyses, participants without migration background had approximately twofold higher odds of diabetes technology use at follow-up than those with migration background (OR 2.21, 95% CI 0.98–4.98, *p* = 0.055). After adjustment for age, sex, and diabetes duration, the magnitude of the association remained similar (OR 2.33, 95% CI 0.92–5.89, *p* = 0.075) (Fig. 2).

Younger age (OR 0.66, 95% CI 0.56–0.79, *p* < 0.001) and longer diabetes duration (OR 1.32, 95% CI 1.14–1.53, *p* = 0.0003) were significantly associated with higher technology use. Sex was not associated with diabetes technology use (Fig. 2).

Firth logistic regression yielded comparable results. Household language was not associated with technology use (adjusted OR 0.88, 95% CI 0.26–3.02, *p* = 0.836).

### Metabolic outcomes

Mean HbA1c at follow up was 7.6% ± 1.5% (60 mmol/mol) in participants without MB and 8.0% ± 1.8% (64 mmol/mol) in those with MB, without a statistically significant difference (*p* = 0.181) (Table 2).

The proportion of participants achieving the HbA1c target ≤ 7.0% (≤ 53 mmol/mol) was 41.7% in the non-MB group and 31.4% in the MB group (*p* = 0.281) (Table 2). No significant differences were observed across migration subgroups.

### Continuous glucose monitoring

At diabetes onset, CGM use did not differ between groups (26.2% vs. 24.2%, *p* = 0.822). At follow-up, CGM use was high in both groups (94.4% vs. 85.7%) (Table 2).

### Acute complications

No significant differences were observed in rates of diabetic ketoacidosis at diagnosis (51.0% vs. 45.0%, *p* = 0.629) or since diabetes diagnosis between groups. Severe hypoglycaemia since diagnosis was rare and did not differ significantly between groups (Table 2).

## Discussion

This single-centre cross-sectional study suggests that children and adolescents with migration background were less likely to receive insulin pump therapy at diabetes diagnosis. These findings are consistent with the hypothesis that disparities may primarily affect the timing of diabetes technology initiation rather than long-term access.

Lower use of insulin pump therapy among children with migration background is consistent with previous studies reporting reduced uptake of diabetes technology in migrant populations [10–12, 15]. For example, Icks et al. showed lower insulin pump use among children with Turkish background in Germany [10], and Agarwal et al. reported marked disparities across ethnic groups in the United States [11].

At follow-up, differences in diabetes technology use were smaller than at diagnosis and were no longer statistically significant. This pattern is consistent with the hypothesis of delayed uptake of diabetes technology, although the cross-sectional design does not allow conclusions regarding the exact timing of technology initiation. If confirmed in longitudinal studies, such a distinction could have important implications for the timing of interventions aimed at improving equitable access to diabetes technology. This is clinically relevant, as early initiation of insulin pump therapy and automated insulin delivery systems has been associated with improved glycaemic outcomes and reduced acute complications [16, 17]. Efforts to minimise delays in technology initiation, particularly in vulnerable populations, are therefore warranted.

In contrast to studies reporting persistent disparities in technology use and metabolic outcomes [10–12, 18–21], differences in diabetes technology use were smaller and no longer statistically significant at follow-up in our cohort. One possible explanation is the Austrian healthcare system, where access to diabetes technologies is largely independent of financial resources and embedded in structured paediatric diabetes care.

The observed pattern may be related to challenges in the early phase after diagnosis, when families are required to rapidly acquire complex disease management skills. Barriers such as health literacy, cultural factors, and unfamiliarity with the healthcare system may particularly affect this initial phase, even in settings with universal healthcare access [13].

In multivariable analyses, adjustment for age, sex, and diabetes duration had little impact on the magnitude of the association between migration background and diabetes technology use, although the confidence interval included the null value. In addition, participants with migration background had a shorter diabetes duration, which was independently associated with diabetes technology use and may partly have contributed to the lower technology use observed at follow-up. Given the limited sample size, the adjusted analyses should be interpreted with caution. Household language was not associated with diabetes technology use, suggesting that barriers may extend beyond language alone and could also include broader social or healthcare system-related factors.

Younger age and longer diabetes duration were independently associated with higher use of diabetes technology, highlighting the importance of clinical factors in technology uptake. Early initiation of insulin pump therapy requires intensive education and active engagement of families, which may be particularly challenging during the acute phase after diagnosis. In some clinical situations, a stepwise introduction of advanced diabetes technologies may therefore represent a pragmatic and appropriate approach. However, delayed technology initiation may also reflect modifiable barriers rather than clinical considerations alone, underscoring the importance of providing targeted support to families during the early phase of diabetes care.

Despite lower insulin pump use at diagnosis, no significant differences were observed in HbA1c, CGM use or acute complications at follow-up. This may reflect the high overall uptake of diabetes technology during follow-up, the structured paediatric diabetes care provided within the Austrian healthcare system, or the limited statistical power of the present study to detect moderate differences.

This study has several limitations. Its cross-sectional design precludes causal inference. Given the exploratory nature of the analyses, no adjustment for multiple comparisons was performed. The relatively small sample size, particularly in the migration background subgroup, limits statistical power and the precision of effect estimates. Residual confounding cannot be excluded. Furthermore, information on parental education, socioeconomic status, health literacy and detailed language proficiency was not available and therefore could not be considered in the analyses.

The findings of this study have implications for diabetes care in children and adolescents with migration background. Within our centre, the majority of patients received diabetes technology over time, suggesting that access to such therapies can be achieved despite potential barriers. However, lower use of insulin pump therapy at diagnosis indicates that disparities may primarily affect the timing of technology initiation. Early introduction of diabetes technology requires intensive education and engagement, which may be particularly challenging for families facing language, cultural, or system-related barriers.

Targeted strategies to support early access to diabetes technology may include structured and culturally sensitive education, provision of information in multiple languages, and sufficient time and resources for technology training.

In conclusion, our findings suggest lower insulin pump use at diagnosis among children with migration background, whereas differences in diabetes technology use at follow-up were smaller and no longer statistically significant. Future longitudinal studies should investigate whether these findings reflect delayed uptake of diabetes technology and identify potentially modifiable barriers during the early phase after diagnosis. Improving equitable early access to diabetes technology may represent an important opportunity to reduce disparities in paediatric diabetes care.

## Supplementary Information

Below is the link to the electronic supplementary material.

Supplementary Material 1

## Data Availability

The datasets generated and analysed during the current study are available from the corresponding author on reasonable request.
