# Supplementary material for: Migration background and diabetes technology use at diagnosis and follow-up in children and adolescents with type 1 diabetes: a single-centre study
Source: Acta Diabetol. 2026 Jul 29;63(10):1749–55. doi: 10.1007/s00592-026-02758-6 (PMC13687193; doi:10.1007/s00592-026-02758-6)
Supplement: Supplementary file 1 [file 592_2026_2758_MOESM1_ESM.docx]

# Completed STROBE Checklist for Cross-Sectional Studies

**Manuscript:** Migration background and diabetes technology use at diagnosis and follow-up in children and adolescents with type 1 diabetes: a single-centre study

| **Item** | **Section/topic** | **STROBE recommendation (short)** | **Reported on page/section** | **Comments for this manuscript** |
| --- | --- | --- | --- | --- |
| 1a | Title and abstract | Indicate the study design using a commonly used term. | Title and Abstract, p. 1 | The title and abstract state that this is a cross-sectional study. |
| 1b | Title and abstract | Provide an informative, balanced summary of methods and findings. | Abstract, p. 1 | The abstract summarises aims, methods, sample size, main results and conclusions. |
| 2 | Background/rationale | Explain the scientific background and rationale. | Introduction, pp. 1–2 | The introduction describes increasing T1D incidence, migration, poorer outcomes and lower technology use in populations with migration background. |
| 3 | Objectives | State specific objectives and any prespecified hypotheses. | End of Introduction, p. 2 | The study objective is stated: to investigate whether migration background is associated with diabetes technology use at diagnosis and follow-up. |
| 4 | Study design | Present key elements of the study design early in the paper. | Methods, Patients and data collection, p. 2; Abstract, p. 1 | The manuscript identifies the study as a single-centre, cross-sectional observational study. |
| 5 | Setting | Describe setting, locations, dates, and relevant periods of recruitment/data collection. | Methods, Patients and data collection, p. 2 | Setting is the paediatric diabetes centre at the Medical University of Innsbruck; recruitment period was January to June 2023. |
| 6 | Participants | Give eligibility criteria and the sources/methods of participant selection. | Methods, Patients and data collection, p. 2 | Children/adolescents aged 4–19 years with T1D duration >=1 year were consecutively enrolled; other diabetes types were excluded; recruitment flow is reported. |
| 7 | Variables | Clearly define outcomes, exposures, predictors, confounders and effect modifiers. | Methods, Patients and data collection, pp. 2–3; Statistical analysis, pp. 2–3 | Migration background, diabetes technology use, insulin pump/AID/MDI, CGM, HbA1c, DKA, severe hypoglycaemia, age, sex and diabetes duration are described. |
| 8 | Data sources/measurement | For each variable of interest, give sources of data and details of assessment/measurement methods. | Methods, Patients and data collection, p. 2 | Clinical data at diabetes onset and study visit are described; migration history and household language were collected using a structured questionnaire and semi-structured interview. |
| 9 | Bias | Describe efforts to address potential sources of bias. | Methods, p. 2; Limitations, p. 5 | Partly addressed through consecutive enrolment, predefined eligibility criteria, adjusted regression and discussion of limitations.  To minimise selection bias, all eligible patients attending the centre during the recruitment period were approached consecutively. Analyses were adjusted for key clinical covariates, and sparse-data bias was assessed using Firth logistic regression. |
| 10 | Study size | Explain how the study size was arrived at. | Methods, p. 2 | The initial sample size calculation was based on the total paediatric type 1 diabetes population cared for at the Department of Pediatrics 1, Medical University of Innsbruck, Austria, which was estimated to include approximately 200 patients, with an expected ratio of approximately 2:1 between patients without and with migration background. Assuming an independent two-sample t-test to compare HbA1c as the primary outcome between children and adolescents with and without migration background, an effect size of 0.5, a two-sided significance level of 0.05, 80% power, and an allocation ratio of 2:1, a total sample size of 144 participants was estimated to be required, including 96 participants without and 48 participants with migration background.  During the recruitment period, 163 patients fulfilled the eligibility criteria, of whom 155 were enrolled and 143 were included in the final analytic sample. Although the number of participants with migration background was lower than initially planned, the study included the large majority of the eligible source population available at the centre during the recruitment period. |
| 11 | Quantitative variables | Explain how quantitative variables were handled in the analyses, including any groupings. | Statistical analysis, pp. 2–3; Methods, p. 2 | Continuous variables are presented as mean +/- SD; age and diabetes duration are used in adjusted models; HbA1c target <=7.0% is reported. |
| 12a | Statistical methods | Describe all statistical methods, including control for confounding. | Statistical analysis, pp. 2–3 | The manuscript describes t-tests, Chi-square/Fisher tests, Cochran-Armitage trend test, multivariable logistic regression adjusted for age, sex and diabetes duration, and Firth logistic regression sensitivity analysis. |
| 12b | Statistical methods | Describe methods used to examine subgroups and interactions. | Methods/Results, pp. 2–4 | Migration subgroups are described and compared descriptively; no interaction analyses are reported |
| 12c | Statistical methods | Explain how missing data were addressed. | Methods, p. 2 | The 12 individuals with unavailable information on migration background had initially agreed to participate but did not complete the questionnaire/interview items required to classify migration background.  As migration background was the key exposure variable, these individuals could not be assigned to the exposure groups and were therefore excluded from the final analytic sample.  All variables included in the regression analyses were complete, and missing values for other reported variables are shown in the manuscript tables. |
| 12d | Statistical methods | For cross-sectional studies, describe analytical methods accounting for the sampling strategy, if relevant. | Methods, p. 2 | Not applicable or no complex sampling; participants were consecutively enrolled from one centre. |
| 12e | Statistical methods | Describe any sensitivity analyses. | Statistical analysis, p. 3; Results, p. 3 | Firth logistic regression was performed as a sensitivity analysis and yielded comparable results. |
| 13a | Participants | Report numbers of individuals at each stage of the study. | Methods, p. 2; Results, p. 3 | The manuscript reports 163 eligible, 8 declined, 155 enrolled, 12 12 excluded because migration background could not be classified, and 143 analysed. |
| 13b | Participants | Give reasons for non-participation at each stage. | Methods, p. 2 | Reasons are reported for exclusion because migration background could not be classified; declined participation is reported without further reason. |
| 13c | Participants | Consider using a flow diagram. | Methods/Results, pp. 2–3 | Numbers are clear in text. A flow diagram is optional; not essential for this small single-centre study. |
| 14a | Descriptive data | Describe participant characteristics and exposures/potential confounders. | Results, Study population, p. 3; Table 1 | Age, sex, diabetes duration and migration background groups are described; Table 1 is referenced. |
| 14b | Descriptive data | Indicate number of participants with missing data for each variable of interest. | Methods, p. 2 | Missing migration background is reported. The 12 individuals with unavailable information on migration background had initially agreed to participate but did not complete the questionnaire/interview items required to classify migration background. As migration background was the key exposure variable, these individuals could not be assigned to the exposure groups and were therefore excluded from the final analytic sample. All variables included in the regression analyses were complete, and missing values for other reported variables are shown in the manuscript tables. |
| 15 | Outcome data | For cross-sectional studies, report numbers or summary measures of outcome events. | Results, pp. 3–4; Table 2; Figures 1–2 | Technology use at diagnosis and follow-up, HbA1c, CGM use and acute complications are reported by migration background. |
| 16a | Main results | Give unadjusted and adjusted estimates with precision. | Results, Multivariable analysis, p. 3; Figure 2 | Unadjusted and adjusted odds ratios with 95% confidence intervals and p-values are reported. |
| 16b | Main results | Report category boundaries when continuous variables are categorised. | Results, Metabolic outcomes, p. 4 | HbA1c target is defined as <=7.0% (<=53 mmol/mol). Migration background categories are defined in Methods. |
| 16c | Main results | If relevant, translate relative risk into absolute risk for a meaningful time period. | Results, pp. 3–4 | Not applicable for this cross-sectional analysis; absolute proportions are reported alongside odds ratios. |
| 17 | Other analyses | Report other analyses, such as subgroup and sensitivity analyses. | Results, pp. 3–4 | Firth sensitivity analysis and household-language model are reported; migration subgroup comparisons are also presented descriptively. |
| 18 | Key results | Summarise key results with reference to study objectives. | Discussion, pp. 4–5 | The discussion summarises lower pump use at diagnosis and attenuated differences at follow-up. |
| 19 | Limitations | Discuss limitations, including potential bias, imprecision and direction/magnitude of bias. | Discussion, Limitations paragraph, pp. 4-5 | The manuscript notes cross-sectional design, limited sample size, residual confounding, limited generalisability and sparse data for region-of-origin analyses. |
| 20 | Interpretation | Provide a cautious overall interpretation considering objectives, limitations, multiplicity and external evidence. | Discussion, pp. 4–5 | Interpretation is cautious and compares findings with previous studies; the conclusion focuses on delayed initiation rather than persistently reduced use. |
| 21 | Generalisability | Discuss the generalisability of the findings. | Discussion, Limitations paragraph, pp. 4-5 | Generalisability is addressed, particularly regarding the distribution of countries of origin and the single-centre Austrian setting. |
| 22 | Funding | Give the source of funding and the role of funders, if applicable. | Funding, p. 5 | Funding source is reported. Consider adding whether the funder had no role in study design, analysis, interpretation or manuscript preparation, if required by the journal. |
